# Neuroathletic training in stroke rehabilitation? A single-blind randomized controlled pilot study on the potential of neuroathletic training for balance ability in stroke outpatient rehabilitation

**DOI:** 10.1186/s13104-024-07022-0

**Published:** 2024-12-12

**Authors:** Judith Evers, Isabel Stolz, Marilena Klein

**Affiliations:** 1 Institute of Movement and Neurosciences, Department Movement Rehabilitation, Neuromechanics and Paralympic Sports (IV), German Sport University Cologne, Am Sportpark Müngersdorf 6, 50933 Cologne, Germany; 2 Oberberg Clinic, RPP Society for Rehabilitation, Prevention & Care mbH, Am Hüttenberg 1, 51643 Gummersbach, Germany

**Keywords:** Medical rehabilitation, Movement therapy, Neurorehabilitation, Stroke rehabilitation

## Abstract

Recently, neuroathletic training has been increasingly applied in competitive sports, in therapy, and in prevention. Scientific evidence on the effectiveness of this approach, however, has been poorly developed. Potentials of neuropathologic perceptual exercises to potentially improve balance control in the context of movement therapy in stroke rehabilitation appear promising. To investigate the possible effects of neuroathletic exercises on the balance ability of poststroke patients with ischemic infarcts and intracerebral hemorrhages, a controlled trial of a standardized protocol of daily neuroathletic exercises compared to traditional movement therapy during a four-week period of medical rehabilitation was conducted (n = 19). Patients were assessed with the Berg-Balance Scale (BBS), which represents the Gold standard for clinical measurement of balance. The results of the prepost BBS measurement showed significant balance improvements in the intervention and control groups, whereas the intervention group reached a higher total score by half the size of the standard deviation. A comparison of the results of both groups supported the general effectiveness of movement therapy for improving balance in stroke rehabilitation. However, neuroathletic training exercises did not lead to a stronger effect. Moreover, the intervention group did not score significantly lower than the control group. A negative influence can therefore not be assumed. For further investigations of neuroathletic training compared to treatment-as-usual training to improve balance in stroke rehabilitation, additional studies with larger sample sizes and longer treatment periods should be conducted.

*Trial registration* United States National Library of Medicine, NCT06391801, date: 04.29.2024.

## Introduction

Based on anecdotal evidence, neuroathletic training is described as effective for target groups in performance sports, leisure sports and movement therapy. Neuroathletic training is being increasingly integrated into practical fields of training, coaching education and therapy [1–3]. In addition to athletic objectives, central nervous system movement control and specific perception exercises, which are included in specific training sessions, are addressed [4]. Although this topic is receiving increased amounts of attention in the literature and from an increasing number of workshops for practitioners, scientific evidence supporting the effectiveness of therapeutic approaches is lacking. Only a few research findings show positive effects in the context of specific neuroathletic exercises [2, 5]. With respect to patients in stroke rehabilitation, exercises to stimulate the visual and vestibular systems were found to be effective for movement rehabilitation [6]. It could thereby be useful to consider the potentials of the approach for the target group post stroke patients and further clinical populations.

The clinical condition stroke itself does not represent a homogeneous, defined clinical picture. Rather, it describes a variety of different circulatory disorders of the brain [7]. Main forms are ischemic infarcts and intracerebral hemorrhages, which can result in years of functional impairments with very different symptoms occurring depending on the localization, affected area and severity [7, 8]. These can manifest themselves in sensorimotor, emotional, cognitive or social deficits [8]. Among other things, hemiparesis, which in around 50% of cases is accompanied by sensory disturbances, cognitive-mnestic symptoms, orientation disorders, apraxia, coordination disorders, vertigo, dysarthrophonia or dysphagia. Rehabilitation measures consist of a variety of different applications, such as speech therapy, psychological counseling, occupational therapy and physiotherapeutic treatments, which are mainly aimed at restoring the patient's motor functions [9, 10]. In this regard, classic concepts such as Bobath, Vojta, proprioceptive neuromuscular fascillation (PNF) and physiotherapy have existed for several decades. Van Cranenburgh [11] notes that some of these concepts are aimed exclusively at improving Activities of Daily Living (ADL) and are therefore less helpful for occupational purposes and leisure activities [11]. According to Lamprecht and Lamprecht [12], traditional concepts are increasingly being replaced “by new evidence-based therapy concepts” such as constraint-induced movement therapy (CIMT) and robot-assisted training including growing approaches of strength training [12].

In neurological rehabilitation in general and stroke therapy in particular, the ability to maintain balance is a mayor rehabilitation goal, since its essential for everyday activities. It represents the basis for sitting, standing and walking and all resulting everyday life activities. Stroke patients experience deficits in balance and losses in motor function during activities of daily living; therefore, temporal precision activity-related stimuli could complement movement therapy to provide targeted support for neuronal plasticity to restore functions over time [13]. Outpatient rehabilitation movement therapy aims to promote physical functions that are important in everyday life, including coordination and balance tasks, to enable independent and safe movement [14, 15].

Exploring neuroathletic perceptual exercises to improve balance seems to be promising for the target groups since a main focus are exercises to work on information processing of the brain and sensorics system [16]. It is essentially based on findings from neuroscientific research and was developed in America including a comprehensive course and training system with various key topics [17]. Neuroathletic training focuses on the quality and processing of the incoming information in the brain and aims to improve the quality of the training through more precise personal risk assessment to improve the quality of movement [16]. The approach involves improving performance in (sporting) movements through improved information intake and processing without having directly influenced classic parameters, such as muscle strength, through traditional training [17]. According to this principle, neuroathletic exercises (so-called drills) for the three systems (visual, vestibular, proprioceptive) are selected and practiced in short training units of 15 to 40 min, based on practical experience [17]. It is also recommended to monitor the effects of the drills at regular intervals [17]. Scientific evidence about effectiveness of the approach exists only to a limited extent.

Two scientific studies can be mentioned, that examined the effects in the context of sport. Di Vico et al. [5] investigated differences due to a change in tongue position during isokinetic knee flexions and extensions and showed significant differences in force development of up to 30% [5]. They attributed this to a connection between the tongue position and the central nervous system, but emphasize that further studies are required to verify this hypothesis [5]. In contexts of college football, Clark et al. [18] investigated the influence of different visual training methods on the frequency of concussions suffered by players compared to previous years without intervention. During the intervention period, the values per season were significantly lower than in the four previous years, but neuroathletic training was applied among other approaches [18]. In the scientific discourse, the approach is viewed critically, as it appears unclear whether the structures targeted by the training structures targeted by the training are actually relevant in terms of performance physiology and whether effects occur where neuroathletes postulate this [19]. The complex processes of neurophysiological information processing may be simplified in the approach, which may not do justice to the complex nature of the processes. Based on studies by Beedie and Foad [20] and Bérdi, Köteles, Szabó and Bárdos [21], potential effects could also occur due to placebo responses [20, 21]. To explore the potential of neuroathletic training for stroke rehabilitation, a single-blinded controlled pilot study was conducted in a stroke outpatient rehabilitation program in this study.

## Methods

### Study design

The study design was a monocentric single-blinded randomized controlled pilot study (n = 19). The aim of the study was to investigate the potential effects of a 4-week outpatient rehabilitation program that included 15 min of daily neuroathletic training compared to 15 min of treatment as usual (traditional movement therapy). Traditional movement therapy was conducted according to German framework recommendations for outpatient neurological rehabilitation [9, 13]. It included physical therapy and ergo therapy in the individual and group setting, which focused exercises on sensorimotor skills, stance and gait motor skills, hand and finger motor skills, training of self-care in everyday life, locomotion in immediate and wider surroundings (see Appendix B). Also, psychosocial skills to improve attention, memory, planning and memory, the ability to plan and to act are part of the neurological outpatient rehabilitation [13]. Treatments generally take place 3 to 5 times a week over several hours on 15 to 20 treatment days [13].

The primary endpoint was balance ability in stroke outpatient rehabilitation, measured via the German version of the Berg balance scale (BBS) before and after the intervention was administered; this was conducted by one blinded therapist during the years 2021 and 2022, the intervention period ran from December 2021 to March 2022. [22–24]. The BBS was chosen because represents the gold standard for measuring balance in a clinical setting, furthermore it enables the results of this therapy to be compared with other interventions [25–27]. The BBS was assessed in all participants on the first day of their rehabilitation program before the start of therapeutic measures. The second and final assessments of the BBS were carried out on the last day after the completion of all the rehabilitation measures. The pilot study was applied to determine feasibility of neuroathletic methods and effect sizes in clinical populations, that may be used subsequently in larger scale studies.

### Population

Included post-stroke patients had the forms of stroke ischemic infarcts or intracerebral hemorrhages. Patients in outpatient rehabilitation were fewer severely affected than patients with inpatient stay and able to walk unassisted. Participants were randomly assigned to the control or intervention group by a coordinating administration employee at the study site via simple concealed randomization. Participant selection took place ad hoc. The inclusion criteria for study participation were a main clinical diagnosis of the two forms of stroke and indication for outpatient rehabilitation, including movement therapy, as well as adequate fitness to participate in a 4-week movement therapy program. All the subjects agreed to participate voluntarily and provided written informed consent. The study was approved by the Ethics Committee of the German Sports University Cologne in accordance with the Declaration of Helsinki (Seventh revision, 2013).

### Study procedure

The neuroathletic training included a structured exercise catalog prepared in advance and performed in a standardized manner for all patients in the intervention group. The conducting therapists received an introduction to carry out neuroathletic training and received all the materials necessary for the intervention as well as the standardized training program with the exercise catalog to perform almost identical training (Table 1, Appendix A). All the other therapeutic procedures were the same for the intervention and control groups. According to Schmid-Fetzer and Lienhard [3], more frequent short training sessions are preferred over a few longer sessions. Therefore, training was conducted daily for 15 min with each patient. The total duration of neuroathletic training in the intervention period was therefore 5 h per patient in the intervention group.

### Measures and statistical analyses

As in the original English version, the German version of the Berg balance scale comprises 14 test items, which can be rated on a 5-point Likert scale ranging from 0 to 4 points. It represents a well-established and valid measure and is considered the Gold standard for assessing balance [25]. The total score is calculated by adding the values of each item and is a maximum of 56 points. According to Berg, Wood-Dauphinee, Williams and Maki [23], people with a total score of less than 45 points are considered to have impaired balance and are at increased risk of falling. The data analysis was carried out via IBM SPSS Statistics Version 28 (190). The demographic data were analyzed via descriptive statistics. The change in the subjects' ability to balance was analyzed in both groups using the Wilcoxon rank test. Differences in the values between the control and intervention groups were analyzed using the Mann‒Whitney U test (significance level α = 5%) due to the small sample size. Effect sizes were calculated via Cohen’s d.

## Results

**Fig. 1:**
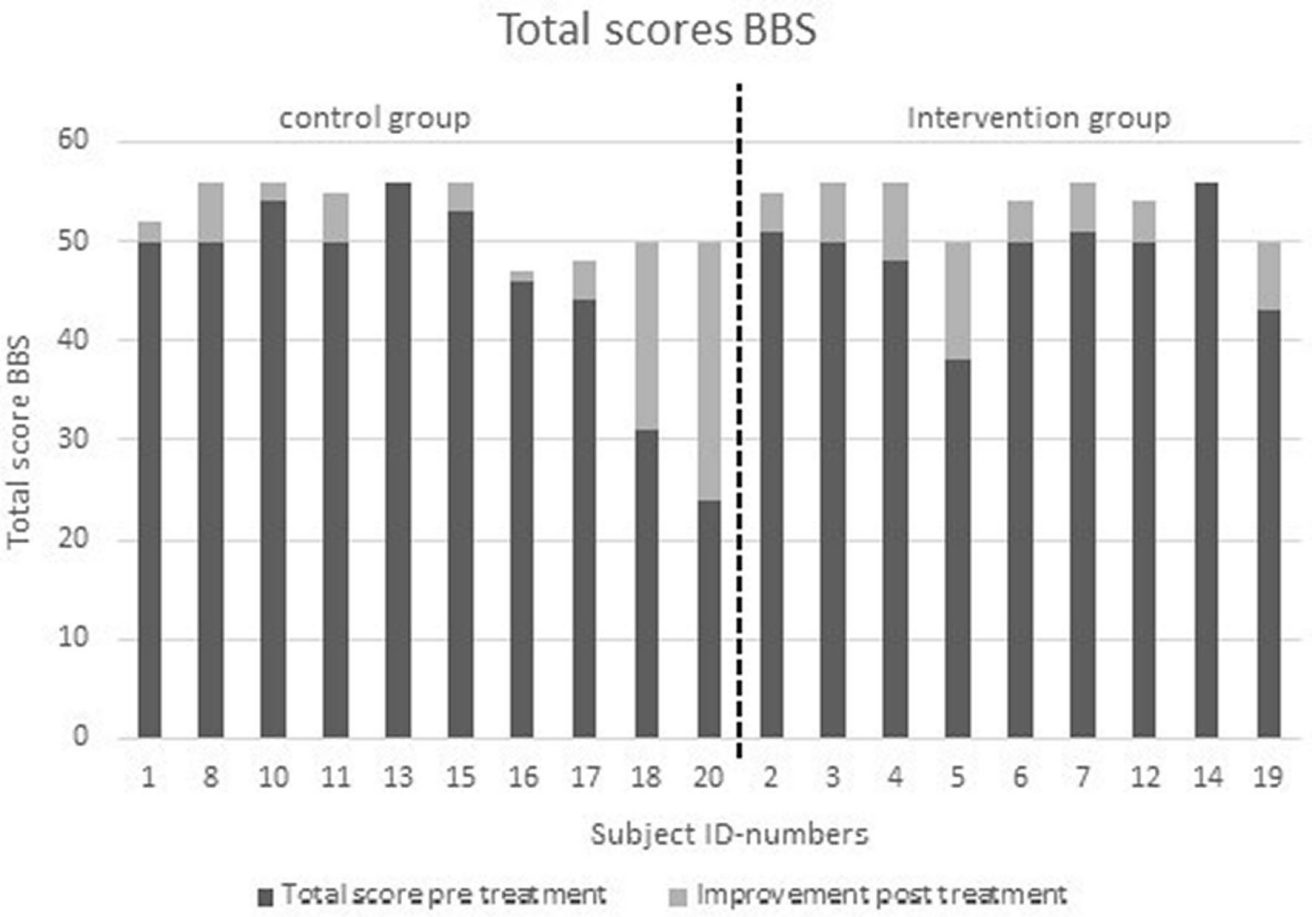
Total scores pre- and post-treatment (n = 19)

Among the initial 20 randomized subjects, 19 successfully completed the study (11 males, 8 females, 1 drop-out). The person, who dropped out, paused the entire outpatient therapy for personal reasons, therefore a continuation of the study could not be realized. The average age of the individuals in the total sample was M = 66.47 years (SD = 10.75, Min = 45.00, Max = 83.00). The intervention group (IG) consisted of three male and six female participants with an age of M = 63.56 years (SD = 11.50, Min = 45.00, Max = 83.00), and the control group (CG) consisted of eight males and two females with an age of M = 69.10 years (SD = 9.86, min. (SD = 9.86, Min = 51.00, Max = 81.00). The results of the pre-post intervention measurements via the Berg balance scale showed significant balance improvements in the intervention and control groups, whereas the intervention group had a total score that was half the standard deviation (pretest IG: M = 48.56, SD =5.20; CG: M = 45.80, SD = 10.40; posttest IG: M = 54.11, SD = 2.47; CG: M = 52.60, SD = 3.63) (see Figure 1).

Group comparisons did not reveal a significant difference between groups (p = .411, effect size d_corr_  = .151). As almost 45% of the patients in the IG reached the maximum scores in the posttest, this complicated the precise comparison of changes among the groups. The control group randomly included more patients with lower test scores in the pretest, which enabled a comparatively greater improvement (minimum of the IG = 38.00; minimum of the KG = 24.00). The standard deviation was also comparatively high in the control group. Here it became apparent that people responded to the traditional treatment as usual measures to varying degree. Especially patient 20 responded comparably high to the treatment, possibly due to a higher fitness level prior to the stroke or a comparatively higher motivation.

## Discussion

A comparison of the BBS scores of both groups showed that the analyses were in concordance with the scientific results confirming the general effectiveness of movement therapy for improving balance in stroke rehabilitation [6]. Neuroathletic training did not lead to a stronger effect. Moreover, the intervention group did not score significantly lower than the control group. A negative influence can therefore not be assumed. Blum and Korner-Bitensky [27] noted possible bottom and ceiling effects in stroke patients when using the BBS, yet the BBS represents a valid method for determining balance in post-stroke rehabilitation [27–29]. For this investigation, it can be concluded that distribution imbalances had an impact on the results. Some patients in the IG already had high scores in the pretesting session and could hardly improve within the BBS scaling. As a result, the potential for improvement in the CG was slightly greater than that in the IG. At the same time, there were also patients with scores less than 45 points; these patients were considered to be at increased risk of falling. A higher test difficulty would no longer have been feasible for them [25]. In future studies, the BBS could be combined with other instruments, such as the Dynamic Gait Index (DGI), in order to assess changes in balance more sensitively when walking, also in highly physically fit patients, who already reached higher scores in the BBS in the pre-measurement [30].

This heterogeneity and distribution of participants were limiting factors within the analyses and should be addressed in future studies to achieve greater homogeneity within the total study population.

The pre-post comparison of the mean values showed significant improvements in balance ability in both groups. Especially participants with the lowest scores in the first measurement were able to improve their scores by the 45-point limit through the rehabilitation measures, thereby significantly reduce their risk of falling and considerably improve their self-determination and participation considerably. Patients in both groups surpassed the minimal clinically important difference (MCID) of four points in Berg Balance Scale scores among patients with stroke, who are able to walk unassisted [31]. Since MCID is applied to assist the clinical determination of the effectiveness of therapy, it can be confirmed that traditional movement therapy was effective in outpatient rehabilitation for the target group [31]. Additional neuroathletic training did not lead to a significantly stronger effect but also did not counteract the treatment effectiveness in regard to MCID. The intensity of the neuroathletic training in this study can be considered quite high, with 15 min of daily training for 4 weeks; however, to depict potential long-term neurological adaptations, the intervention period and intensity were not sufficient. According to the authors, an effect should be achieved after 10 min of daily training. A total of 25 to 30 total hours of neuroathletic training as total therapeutic volume are recommended by Schmid-Fetzer and Lienhard [3]. Therefore, a limitation of the study is the limited intervention time, which could be extended in future investigations. However, an expansion of neuroathletic training in therapy would inevitably be associated with a reduction in other established and evidence-based therapeutic interventions, which should be considered carefully. As researchers critically discuss whether a neuroathletic training stimulus actually causes a sustainable change and enables an effect where it is considered relevant, suitable study designs should be given careful consideration [19].

In further investigations it would be beneficial to distinguish the effects between the interventions more clearly, for example by means of randomized controlled trials with cross-over designs or more in-depth and extended intervention periods, also combined with objective neurophysiological measurement technology, such as Electroencephalography (EEG). It should also be investigated in greater depth what causes varying degrees of response to the therapy in a diverse group of patients.

Furthermore, other movement therapeutic approaches based on the improvement of neuronal processes appear to be scientifically more evident for post-stroke balance rehabilitation and more successful in therapeutic practice, such as previously stated CIMT [32]. Further studies could rather follow up on these evidence-based approaches in depth and apply them to different target groups to create most suitable protocols for the target group, considering that patients can only invest a certain amount of time and energy in the rehabilitation process.

Overall, further and more extensive research is needed to clarify the effectiveness of neuroathletic training and therapy approaches in competitive, recreational and health training as well as in a rehabilitative context to create sufficient scientific evidence and to advise patients on effective and appropriate interventions against the background of their medical condition.

A further limitation of the pilot study is the small sample size, which did not allow parametric analyses. For further investigation, additional studies with larger sample sizes should be conducted to adequately determine the potential underlying effects of these interventions. Thus, additional empirical evidence on the actual usefulness of neuroathletic intervention contents can be generated subsequently.

## Conclusion

To investigate effects of neuroathletic training exercises on the balance ability of poststroke patients, a randomized controlled trial was applied in outpatient rehabilitation in this study. Included patients completed a 15-min standardized protocol of daily neuroathletic exercises additional to their regular movement therapy compared to patients participating in traditional movement therapy during a four-week period of medical rehabilitation. Advantages of neuroathletic training compared to treatment-as-usual for improving the balance in people with stroke could not be confirmed in this pilot study. Both groups showed significant improvements in balance ability with a reduced risk of falling, merely the standard deviation (SD) was also comparatively low in the intervention group. In conclusion, it should be pointed out that despite of the limitations in this pilot study, a comparatively high intensity with daily neuroathletic exercises could be realized over a period of 4 weeks. The study design contained a reduction of confounding variables and distortions, such as investigator blinding and control group design, as well as application in a relevant target group, yet no significant differences to regular outpatient rehabilitation effects were achieved. In light of the current state of scientific research, the results point to a critical view of the neuroathletic approach as a superior training concept for the target group in addition to traditional movement therapeutic treatment in outpatient rehabilitation.

## Data Availability

No datasets were generated or analysed during the current study.

## Appendix A

See Table 1

**Table 1:** Neuroathletic exercise protocol*

standardized training exercises
1) Visual head movement NO/NO Exercise (fixating visual points at eye level, above eye level, below eye level and exercise with closed eyes)
2) Visual head movement Yes/Yes Exercise (visual points centered, points left of center, points right of center, with closed eyes) lateral head tilt (open eyes, closed eyes)
3) Vestibulo-ocular reflex (VOR) (Simplified entry, basic exercise)
4) VOR-C as full body rotation (reduced target (to the right, to the left), simplified entry, partial rotations (to right and left) complete rotations (to right and left)
5) Various balance forms, head is held still (eyes open, eyes closed)
6) Various balance forms with head movement (eyes open, eyes closed)
7) Thumbs follow (basic exercise, exercise with reduced visual target)
8) VOR (Advanced) (way back with eyes closed)
9) Teeter on gymnastics ball (visual target at eye level, head to the side tilted (static), head turned to the side (static), eyes closed, head tilted to the sides, (dynamic) head turned to the sides, (dynamic)
10) Lunge with bar (open eyes (tilt angle reduced), open eyes (tilt angle 45°), closed eyes (tilt angle 45°)
11) Compass steps (holding while standing, free standing, varying movement amplitudes)
12) Course of eight with and without visual target (forward, backward)
13) Teeter with visual target (moderate amplitude of motion, large amplitude of motion)
14) Walking with visual target (small steps, large steps, walking with higher speed, walking with laterally tilted head)

*All the exercises involved participants sitting, standing, standing while holding, working in a wide stand, neutral stand, narrow stand or tandem stand, except for the exercises performed with teeter on gymnastics balls, lunges with bars, compass steps, teeter with visual targets and walking with visual targets

## Appendix B

See Table 2

**Table 2:** Traditional movement therapy

traditional exercises for stroke rehabilitation (exercise excerpt)
1) Standing on one leg unsupported
2) Two-legged stand or one-legged stand with head movement
3) Two-legged stand or one-legged stand with closed eyes
4) Two-legged stand or one-legged stand with additional movements of the upper extremities
5) Balance exercises using the therapeutic device Posturomed^®^ and/or AIREX^®^ Balance-pad
6) Walking straight on a line on the floor
7) Shifting body positions
8) Tactile pressure of the therapist's hand on the upper body and back to train the maintenance of balance in a standing position
9) Climbing over various objects on the floor (with or without support)
10) Fine motor tasks, such as tying a knot or putting the cup upside down and back (supination/pronation exercises)
11) Rope pulling exercises—simultaneous pulling right/left—Hemiparesis: training of the affected side in the upper extremities, same applies to affected side of lower extremities
12) gait training on the treadmill (walking forwards, backwards and sideways), correct rolling 'across the entire foot)
13) Physiotherapeutic treatment techniques (regulating muscle tone, Bobath therapy, Mc Kinsey)
14) Hemiparesis: back treatments for an improvement of symmetry
