# Correction: Neuroathletic training in stroke rehabilitation? A single-blind randomized controlled pilot study on the potential of neuroathletic training for balance ability in stroke outpatient rehabilitation

**DOI:** 10.1186/s13104-025-07415-9

**Published:** 2025-09-04

**Authors:** Judith Evers, Isabel Stolz, Marilena Klein

**Affiliations:** 1 Institute of Movement and Neurosciences, Department Movement Rehabilitation, Neuromechanics and Paralympic Sports (IV), German Sport University Cologne, Am Sportpark Müngersdorf 6, 50933 Cologne, Germany; 2 Oberberg Clinic, RPP Society for Rehabilitation, Prevention & Care mbH, Am Hüttenberg 1, 51643 Gummersbach, Germany

**BMC Research Notes (2024) 17:358**

10.1186/s13104-024-07022-0

Following publication of the original article [1] we were informed that the authors’ given and family names had inadvertently been interchanged.

The correct author names are Judith Evers, Isabel Stolz and Marilena Klein.

The correct author names are shown in the author list of this Correction. The original article has been updated.
